# Aqueous Extract from Pepino (*Solanum muricatum* Ait.) Attenuated Hyperlipidemia and Cardiac Oxidative Stress in Diabetic Mice

**DOI:** 10.5402/2012/490870

**Published:** 2012-07-08

**Authors:** Zhi-hong Wang, Cheng-chin Hsu, Mei-chin Yin

**Affiliations:** ^1^School of Nutrition, Chung Shan Medical University, Taichung 404, Taiwan; ^2^Department of Nutrition, Chung Shan Medical University Hospital, Taichung 404, Taiwan; ^3^Department of Nutrition, China Medical University, Taichung 404, Taiwan

## Abstract

This study examined the lipid-lowering and cardiac protective effects of aqueous extract of pepino (*Solanum muricatum* Ait.) in type 2 diabetic mice. Pepino at 1, 2, or 5% was supplied for 8 weeks. Results showed that pepino significantly decreased water intake and epididymal fat pad weight in diabetic mice (*P* < 0.05). Pepino treatments also significantly reduced plasma glucose and insulin levels, HOMA-IR index, and improved oral glucose tolerance (*P* < 0.05). Plasma and hepatic levels of triglyceride and total cholesterol (TC) were higher in diabetic groups when compared with normal group (*P* < 0.05), pepino treatments at 2 and 5% decreased triglyceride and TC levels in both plasma and liver (*P* < 0.05). Diabetes enhanced mRNA expression of resistin and diacylglycerol acyltransferase1 (DGAT1) in epididymal fat pad (*P* < 0.05); however, pepino intake significantly suppressed mRNA expression of resistin and DGAT1 in epididymal fat pad (*P* < 0.05). Pepino intake significantly reduced reactive oxygen species level, increased glutathione level, and retained glutathione peroxidase and catalase activities in cardiac tissues (*P* < 0.05). These findings suggest that pepino could be considered as a functional food for the alleviation of type 2 diabetes.

## 1. Introduction

Diabetes mellitus (DM) is a chronic metabolic disease. Hyperlipidemia, atherosclerosis, and even diabetic cardiomyopathy are import pathogenic characteristics of DM [1, 2]. The progression of these disorders definitely leads to organs' malfunction and raised morbidity and mortality of DM. It is known that excessive accumulation of lipid such as triglyceride and cholesterol in circulation and organs due to insulin resistance is a major cause response for the occurrence of hyperlipidemia and atherosclerosis in DM patients [3, 4]. On the other hand, oxidative stress from hyperglycemia enhances the production of reactive oxygen species (ROS) and promotes the impairment of organs, which facilitates DM deterioration [5, 6]. Thus, in order to prevent or delay the development of diabetic complications, dyslipidemia and oxidative injury in circulation and organs should be carefully monitored and controlled.

 Pepino (*Solanum muricatum *Ait.) is a plant food in Taiwan. It is well known for its antitumor effects against prostate, stomach, liver, and breast cancer cell lines via its cytotoxic activity [7]. Our previous study found this plant food contained several phenolic acids and flavonoids, and the intake of its aqueous extract markedly improved glycemic control, and mitigated renal oxidative, inflammatory, and glycative stress via reducing ROS and cytokines production, restoring glutathione peroxidase (GPX) activity and declining aldose reductase activity in type 1 diabetic mice [8]. However, it remains unknown that this plant food could provide hypolipidemic and cardiac protective effects under diabetic condition. Furthermore, in order to enhance the possibility of using pepino against diabetes, a type 2 diabetes animal study was designed to examine the effects of pepino upon dyslipidemia and cardiac protection. 

Resistin is a signal molecule secreted from the adipocytes in rodents. It also plays an important role in glucose homeostasis and is related to insulin resistance in the progression of type 2 DM and other metabolic disorders in human [9, 10]. Since pepino aqueous extract could improve glycemic control in our previous study [8], it is reasonable to hypothesize that pepino may regulate resistin and raise insulin sensitivity. In addition, diacylglycerol acyltransferase1 (DGAT1) catalyzes the final step in the synthesis of triacylglycerol from diacylglycerol and fatty acyl-coA. The increased expression of this enzyme promotes excessive lipid biosynthesis and deposit [11]. It is reported that this enzyme is highly correlated with pathogenesis of obesity and type 2 DM [12]. Thus, any agent with the potent to suppress this enzyme may be able to decrease lipid accumulation in organs.

In this study, high-fat diet combined with low-dose streptozotocin (STZ) was used to induce type 2 DM in mice. The effects of aqueous extract from pepino upon hyperlipidemia, cardiac oxidative injury, resistin, and DGAT1 regulation in diabetic mice were examined to further understand the antidiabetic activities of pepino.

## 2. Materials and Methods

### 2.1. Materials

Fresh pepino (*Solanum muricatum *Ait.) was obtained from farms in Penghu island, Taiwan. A 50 g edible portion of pepino was chopped and mixed with 150 mL sterile distilled water followed by homogenizing in a Waring blender. After filtration through Whatman no. 1 filter paper, the filtrate was further freeze-dried to a fine powder. 

### 2.2. Animals and Diets

Male Balb/cA mice, 5 weeks old, were obtained from National Laboratory Animal Center (National Science Council, Taipei City, Taiwan). The use of mice was reviewed and approved by Chung Shan Medical University Animal Care Committee. To induce type 2 diabetes, we used the method as described by Srinivasan et al. [13], in which mice were fed either a normal chow diet or a high-fat diet consisting of 60% fat. After the initial period of 2 weeks on either group, animals were injected once intraperitoneally with STZ (40 mg/kg BW in 0.1 mol/L citrate buffer, pH 4.5). The blood glucose level was monitored on day 14 after STZ injection using a one-touch blood glucose meter (Lifescan Inc., Milpitas, CA, USA). Mice with fasting blood glucose levels ≥12.0 mmol/L were used for this study. After diabetes was induced, mice were divided into five groups (10 mice per group): diabetic mice with chow diet, or 1, 2, or 5% pepino extract. Body weight, feed intake, and water intake were recorded. One group of nondiabetic mice with normal chow diet and without STZ injection was used for comparison.

### 2.3. Experimental Design

After 8 weeks of pepino administration, an oral glucose tolerance test (OGTT) was performed after a fast of 4 h. Blood samples were obtained from the tail vein to monitor blood glucose levels at 0, 30, 60, and 120 min after oral glucose administration, 2 g/kg BW. Mice were killed with carbon dioxide. Heart, liver, and epididymal fat pad from each mouse were collected. Blood was also collected, and plasma was separated immediately. A 0.1 g sample of heart, liver, and epididymal fat pad were homogenized on ice in 2 mL phosphate-buffered saline (PBS, pH 7.2). The protein concentration of sample homogenate was determined by the method of Lowry et al. [14] using bovine serum albumin as a standard. In all experiments, the sample was diluted to a final concentration of 1 g protein/L. 

### 2.4. Blood Glucose and Insulin Analysis

The plasma glucose level (mmol/L) was measured by a glucose HK kit (Sigma, St Louis, MO, USA). The plasma insulin level (*μ*g/L) was measured by a double-antibody radioimmunoassay method using a rat insulin RIA kit (Millipore, Billerica, MA, USA). Insulin resistance was estimated using the homeostasis model assessment (HOMA-IR) formula: (fasting glucose × fasting insulin/22.5). 

### 2.5. Determination of Lipid Profiles in Plasma and Liver

Concentrations of total cholesterol (TC) and triglyceride in plasma were determined by triglycerides/GB kit and cholesterol/HP kit, (Boehringer Mannheim, Mannheim, Germany), respectively. Lipids were extracted from the liver by the method of Bligh and Dyer [15]. Commercial kits were used to measure concentrations of triglyceride and TC in the liver lipid extract and plasma.

### 2.6. Determination of Antioxidant Status in Heart

Cardiac glutathione (GSH) concentration (nmol/mg protein) was determined by commercial colorimetric GSH assay kit (OxisResearch, Portland, OR, USA). Cardiac activity of GPX and catalase was determined by GPX and catalase assay kits (Calbiochem, EMD Biosciences, Inc., San Diego, CA, USA). The method described in Gupta et al. [16] was used to measure the amount of ROS in heart.

### 2.7. Real-Time Polymerase Chain Reaction (RT-PCR) of Resistin and DGAT1

RT-PCR was performed to quantify the mRNA expression level of resistin and DGAT1 in adipose tissue. Total RNA was extracted using TRIzol reagent (Invitrogen, Life Technologies, Carlsbad, CA, USA) according to the manufacturer's instructions. Total cDNA was obtained by reverse transcription. Briefly, the reaction mixture contained 2 *μ*g total RNA, 2 *μ*L oligo(dT), 2 *μ*L dNTPs (2.5 mmol/L each), 2.5 units of Taq DNA polymerase, and 3.5 *μ*L RNase-free double-distilled H_2_O was added. Then the reaction mixture was heated to 42°C for 120 min and then denatured at 95°C for 5 min. Specific primers are shown in Table 1, and GAPDH was used as the house keeping gene to normalize the values obtained for transcripts under examination. The reaction mixture was incubated at 95°C for 10 min and then run for 40 cycles at 95°C for 15 sec and 60°C for 1 min in the ABI Prism 7000 sequence detection system (Applied Biosystems, Foster City, CA, USA). 

### 2.8. Statistical Analyses

The effect of each measurement was analyzed from 10 mice (*n* = 10). Results were expressed as means ± SD. Statistical analysis was done using one-way analysis of variance, and post hoc comparisons were carried out using Dunnett's *t*-test. Statistical significance is defined as *P* < 0.05. 

## 3. Results

**Figure 1:**
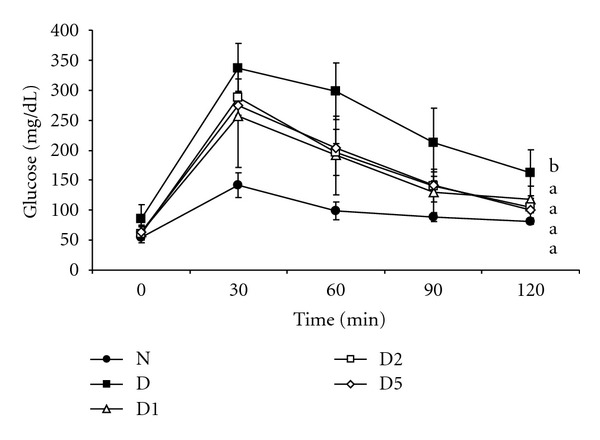
OGTT in normal (N), diabetic mice consuming normal diet (D), or 1% (D1), 2% (D2), 5% (D5) pepino at week 8. Values are represented as mean ± SD (*n* = 10). ^a-b^Means in a certain time point without a common letter differ, *P* < 0.05.

As shown in Table 2, pepino supplement slightly, not significantly, decreased body weight (*P* > 0.05) and significantly lowered water intake and epididymal fat pad weight when compared with diabetic control group (*P* < 0.05). Plasma levels of glucose and insulin increased after the induction of type 2 DM, so did HOMA-IR index (Table 3, *P* < 0.05). Pepino treatments significantly reduced plasma glucose and insulin levels, and HOMA-IR (*P* < 0.05). Pepino intake also improved oral glucose tolerance (Figure 1, *P* < 0.05).

**Figure 2:**
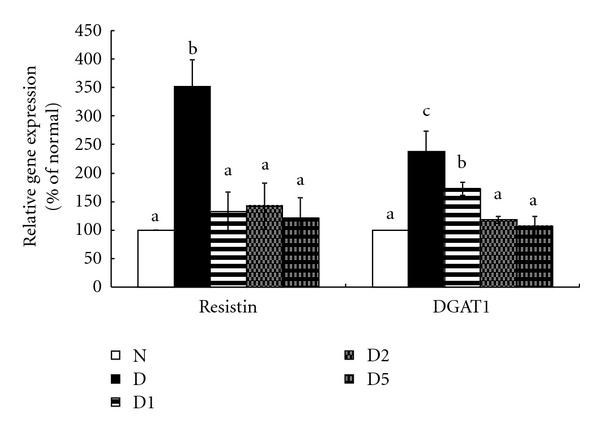
Resistin and DGAT1 gene expressionin epididymal fat pad of normal (N), diabetic mice consuming normal diet (D), or 1% (D1), 2% (D2), 5% (D5) pepino at week 8. Values are represented as mean ± SD (*n* = 10). ^a–c^Means among bars without a common letter differ, *P* < 0.05.

As shown in Table 4, plasma and liver levels of triglyceride and TC were higher in diabetic groups when compared with normal group (*P* < 0.05), pepino treatments at 2 and 5% decreased triglyceride and TC levels in both plasma and liver (*P* < 0.05). Diabetes enhanced the expression of resistin and DGAT1 in epididymal fat pad (*P* < 0.05); however, pepino intake significantly suppressed mRNA expression of resistin and DGAT1 in epididymal fat pad (*P* < 0.05, Figure 2). Pepino treatments significantly reduced ROS level and retained GSH level and GPX and catalase activities in cardiac tissues when compared with diabetic control group (*P* < 0.05, Table 5). 

## 4. Discussion

Our previous study found that supplement of pepino effectively attenuated hyperglycemia and mitigated renal oxidative and glycative injury in type 1 diabetic mice [8]. The results of our current study further revealed that pepino treatment improved hyperglycemia and hyperinsulinemia, reduced cardiac oxidative stress and lipid accumulation in circulation and tissues, and regulated resistin and DGAT1 in type 2 diabetic mice. These findings suggested that pepino could delay the progression of both type 1 and type 2 DM via multiple actions, and its protection was at molecular level because pepino mediated resistin and DGAT1 expression.

Insulin resistance, an important pathological feature of type 2 DM, not only affects glucose and lipid metabolism but also promotes diabetic deterioration [17]. Resistin released from adipose tissue is an important contributor for insulin resistance in peripheral tissue in rodents [18]. Increased serum resistin level in patients with type 2 DM was also reported [19]. Although resistin seems not highly correlated to insulin resistance in human, it plays an important role in DM-associated inflammatory reactions [20]. Our present study found pepino treatment reduced resistin expression in adipose tissue, which subsequently lowered resistin release and finally benefited insulin sensitivity. These results explained the observed alleviated hyperglycemia and hyperinsulinemia in pepino-treated diabetic mice. Our oral glucose tolerance and HOMA-IR data also agreed that pepino intake enhanced insulin sensitivity. These findings implied that pepino could improve glycemic control, and it was partially due to pepino affecting resistin in adipose tissue. Further study is necessary to investigate the impact of pepino upon insulin resistance and/or other inflammation-associated disorders in human subjects.

The most common lipid abnormalities in diabetes are hypertriglyceridemia, hypercholesterolemia, and TG accumulation in liver and adipose tissue. Hyperlipidemia is highly associated with metabolic disorders such as insulin resistance and glucose intolerance [21]. Hyperlipidemia also favors the development of atherosclerosis and diabetic cardiomyopathy [22]. Our present study found that pepino treatment effectively restored insulin sensitivity, which might in turn decrease lipid biosynthesis and deposit in circulation and tissues. Thus, the lower triglyceride and TC levels in plasma, liver, and epididymal fat pad in pepino-treated mice could be partially ascribed to this plant food already restoring the peripheral insulin sensitivity. In addition, we notified that pepino intake markedly downregulated DGAT1, a key enzyme in the synthesis of triglycerides, in adipose tissue. It is reported that the inhibition of DGAT1 is a promising strategy for the treatment of obesity and type 2 diabetes in order to attenuate excessive lipid biosynthesis and deposit [23, 24]. Thus, the suppressive action from pepino upon DGAT1 expression in epididymal fat pad directly explained the less epididymal fat in pepino-treated mice. These findings suggest that pepino could exhibit lipid-lowering activities in circulation, liver, and adipose tissue, which benefited the control of diabetes and/or obesity. 

Heart is a vulnerable organ under diabetic condition. As reported by others [25, 26] and our present study, diabetic mice had substantial cardiac oxidative stress. However, papino supplement effectively mitigated cardiac oxidative stress via reducing ROS formation, sparing GSH level, and retaining GPX and catalase activities. These findings suggest that pepino might protect heart against diabetes-associated oxidative injury, which consequently benefits cardiac functions. Since both hyperlipidemia and cardiac oxidative stress had been ameliorated, the risk of developing diabetic cardiomyopathy was declined in those diabetic mice. It is indicated that phytochemicals from plant foods such as vegetables and fruits may provide multiple protection for DM patients against diabetic deterioration [27, 28]. Our previous study indicated that pepino contained polyphenols including caffeic acid, cinnamic acid, coumaric acid, ellagic acid, ferulic acid, rosmarinic acid, epicatechin, myricetin, naringenin, quercetin, and rutin [8]. It is highly possible that these components contributed to the observed antioxidative and/or lipid-lowering protection in present study. 

In conclusion, our current study demonstrated that aqueous extract of pepino improved insulin sensitivity and glycemic control, as well as attenuated cardiac oxidative stress in type 2 diabetic mice. Pepino also effectively suppressed mRNA expression of DGAT1 and alleviated lipid abnormalities in circulation and tissues. These findings suggest that pepino could be considered as a functional food for the prevention of type 2 diabetes.

## Figures and Tables

**Table 1:** Primer sets used for RT-PCR.

Genes	Forward	Reverse
DGAT1	5^′^-GGTGCCCTGACAGAGCAGAT-3^′^	5^′^-CAGTAAGGCCACAGCTGCTG-3^′^
resistin	5^′^-AGACTGCTGTGCCTTCTGGG-3^′^	5^′^-CCCTCCTTTTCCTTTTCTTCCTTG-3^′^
GAPDH	5^′^-TGTGTCCGTCGTGGATCTGA-3^′^	5^′^-TTGCTGTTGAAGTCGCAGGAG-3^′^

**Table 2:** Body weight (BW, g/mouse), food intake (FI, g/day/mouse), water intake (WI, mL/day/mouse), and epididymal fat pad weight (mg/mouse) of normal (N), diabetic mice consuming normal diet (D), or 1% (D1), 2% (D2), 5% (D5) pepino at week 8.

	N	D	D1	D2	D5
BW	27.5 ± 1.7^a^	32.2 ± 1.7^b^	30.1 ± 2.4^b^	30.0 ± 1.6^b^	30.4 ± 2.5^b^
FI	3.58 ± 0.10^a^	4.54 ± 0.19^b^	4.38 ± 0.37^b^	4.52 ± 0.88^b^	4.13 ± 0.17^b^
WI	2.90 ± 0.14^a^	4.70 ± 0.71^b^	3.50 ± 0.24^a^	3.33 ± 0.47^a^	3.16 ± 0.24^a^
Epididymal fat pad weight	30.0 ± 4.70^a^	49.6 ± 7.00^b^	35.0 ± 9.40^a^	33.5 ± 6.50^a^	32.9 ± 8.50^a^

Value are represented as mean ± SD (*n* = 10). ^a-b^Means in a row without a common letter differ, *P* < 0.05.

**Table 3:** Plasma level of glucose (mmol/L), insulin (*μ*g/L), and HOMA-IR index in normal (N), diabetic mice consuming normal diet (D), or 1% (D1), 2% (D2), 5% (D5) pepino at week 8.

	N	D	D1	D2	D5
Glucose	7.70 ± 0.72^a^	14.60 ± 4.08^c^	12.09 ± 3.51^b^	11.81 ± 1.91^b^	11.65 ± 2.05^b^
Insulin	1.21 ± 0.62^a^	2.10 ± 0.91^b^	1.41 ± 0.76^a^	1.33 ± 0.52^a^	1.22 ± 0.42^a^
HOMA-IR index	11.09 ± 5.67^a^	37.85 ± 6.93^c^	20.61 ± 4.58^b^	16.15 ± 5.99^a^	14.42 ± 5.40^a^

Value are represented as mean ± SD (*n* = 10). ^a–c^Means in a row without a common letter differ, *P* < 0.05.

**Table 4:** Triglycerides (TG) and total cholesterol (TC) levels in plasma (mg/dL) and liver (mg/g protein) of normal (N), diabetic mice consuming normal diet (D), or 1% (D1), 2% (D2), 5% (D5) pepino at week 8.

	N	D	D1	D2	D5
Plasma					

TG	86.68 ± 9.30^a^	138.67 ± 17.98^c^	111.64 ± 7.58^b^	105.51 ± 9.69^b^	88.95 ± 10.24^a^
TC	122.74 ± 5.57^a^	161.21 ± 7.59^b^	152.61 ± 11.59^b^	124.48 ± 6.72^a^	123.62 ± 9.03^a^

Liver					

TG	116.56 ± 13.38^a^	149.61 ± 10.66^b^	119.17 ± 17.89^a^	121.76 ± 15.45^a^	114.51 ± 17.03^a^
TC	30.68 ± 5.52^a^	47.80 ± 6.51^b^	42.16 ± 5.24^b^	36.32 ± 6.07^a^	35.42 ± 4.26^a^

Value are represented as mean ± SD (*n* = 10). ^a–c^Means in a row without a common letter differ, *P* < 0.05.

**Table 5:** Cardiac level (nmol/mg protein) of ROS and GSH, activity (nmol/min/mg protein) of GPX and catalase in normal (N), diabetic mice consuming normal diet (D), or 1% (D1), 2% (D2), 5% (D5) pepino at week 8.

	N	D	D1	D2	D5
ROS	0.47 ± 0.11^a^	1.30 ± 0.17^d^	0.99 ± 0.05^c^	0.91 ± 0.07^c^	0.66 ± 0.14^b^
GSH	11.53 ± 1.11^b^	7.78 ± 0.46^a^	8.26 ± 0.57^a^	8.78 ± 0.89^a^	10.77 ± 0.73^b^
GPX	342.6 ± 30.9^c^	223.0 ± 28.4^a^	252.2 ± 35.2^a^	302.6 ± 18.5^b^	309.3 ± 25.5^b^
Catalase	236.9 ± 19.7^c^	125.9 ± 25.1^a^	149.7 ± 22.7^a^	190.5 ± 17.4^b^	186.8 ± 21.3^b^

Value are represented as mean ± SD (*n* = 10). ^a–d^Means in a row without a common letter differ, *P* < 0.05.
